# SABR-Dual: a phase II/III trial of two-fraction versus five-fraction stereotactic radiotherapy for localized low- and favorable intermediate-risk prostate cancer

**DOI:** 10.1186/s12885-024-12165-1

**Published:** 2024-04-08

**Authors:** Elisha Fredman, Oded Icht, Assaf Moore, Dimitri Bragilovski, Jonathan Kindler, Shay Golan, Dror Limon

**Affiliations:** 1 Department of Radiation Oncology, Davidoff Cancer Center, Rabin Medical Center, 39 Ze’ev Jabotinsky St, Petah Tikvah, Israel; 2 Department of Urology, Rabin Medical Center, 39 Ze’ev Jabotinsky St, Petah Tikvah, Israel

**Keywords:** Prostate cancer, Stereotactic radiotherapy, SABR, Hydrogel spacer, Clinical trial

## Abstract

**Background:**

Dose-escalated radiotherapy is known to improve progression free survival in patients with localized prostate cancer, and recent advances have led to the standardization of ultrahypofractionated stereotactic ablative radiotherapy (SABR) delivered in just 5-fractions. Based on the known effectiveness of the accepted though invasive 2-fraction treatment method of high-dose-rate brachytherapy and given the ubiquity of prostate cancer, a further reduction in the number of treatments of external-beam SABR is possible. This study aims to evaluate the safety, efficacy, and non-inferiority of generalizable 2-fraction SABR compared to the current 5-fraction regimen.

**Methods:**

502 patients will be enrolled on this phase II/III randomized control trial. Eligible patients will have previously untreated low- or favorable intermediate-risk adenocarcinoma of the prostate. Patients will be randomized between standard SABR of 40 Gy in 5 fractions given every-other-day and 27 Gy in 2 fractions at least two days apart but completing within seven days. MRI-based planning, radiopaque hydrogel spacer insertion, and fiducial marker placement are required, and SABR will be delivered on either a standard CT-guided linear accelerator or MR-LINAC. The primary endpoint will be freedom from disease progression, with additional secondary clinical, toxicity, and quality of life endpoints.

**Discussion:**

This study will be the largest prospective randomized trial, adequately powered to demonstrate non-inferiority, comparing 2-fraction SABR to standard 5-fraction SABR for localized prostate cancer. As the protocol does not obligate use of an MRI-LINAC or other adaptive technologies, results will be broadly generalizable to the wider community.

**Trial registration:**

This trial is registered on Clinicaltrials.gov: ClinicalTrials.gov Identifier: NCT06027892.

## Background

Following publication of multiple prospective clinical trials demonstrating the biochemical progression free survival (bPFS) benefits, and possible metastasis-free and cancer-specific survival, of high dose radiation for the curative treatment of localized prostate cancer, typical external beam radiotherapy (EBRT) courses ranged from 38 to 45 daily fractions delivered over 7.5-9 weeks [1–5]. In recent years monumental advances in EBRT have been achieved, allowing significant shortening of the standard radiotherapy course [6–8], and together with diagnostic and technological developments, ultrahypofractionated regimen in the form of stereotactic ablative radiation therapy (SABR) is now an accepted standard of care for definitive treatment in the low- and favorable intermediate-risk settings [9].

At both the individual and systems levels, efficient delivery of patient care and prudent utilization of medical resources is at the forefront. Given the ubiquity of prostate cancer and high patient volumes worldwide, there remains meaningful potential for improvements in patient throughput and cost of care. Presently, the shortest treatment courses for low- and favorable intermediate-risk prostate cancer are radical prostatectomy and low-dose-rate (LDR) brachytherapy, which offer an excellent likelihood of cure [10–11] but are invasive procedures that may not be appropriate, feasible, or desirable for all patients, and include additional costs associated with operating room facilities and anesthesia. A comparable radiation therapy treatment course would help address many of these concerns in patient care.

A strong and mature precedent for a two-fraction course of high dose radiation can be found in the realm of high-dose-rate (HDR) brachytherapy. Under anesthesia, a radioactive source is programmed to traverse catheters that have been transperineally inserted into the prostate in order to produce a desired dose. bPFS rates range between 91 and 97% in the modern literature [12]. While attempts were made to deliver single-fraction HDR brachytherapy, trials consistently revealed the need for two fractions/procedures in the definitive setting [13]. While LDR brachytherapy is technically a single procedure of radiation delivery, the gradual decay of the standard radioisotopes (I-125, Pd-103, Cs-131) over weeks to months is more akin to extreme hyperfractionation over time which may contribute to prolonged toxicity.

There exists, to date, limited data on the safety and effectiveness of prostate SABR delivered in fewer than five fractions. A recent proof-of-concept study by Greco et al. compared single-dose SABR of 24 Gy to 5 fractions of 9 Gy in favorable intermediate and unfavorable intermediate risk patients [14]. They utilized an air-filled rectal balloon to limit rectal motion and planned with no posterior planning target margin. The results demonstrated comparable four-year PSA outcomes for favorable intermediate disease though inferior PFS for unfavorable intermediate disease. Objective and patient reported GU and GI toxicities were not significantly different, though one of the 15 patients in the single-fraction arm experienced delayed grade 3 urethral stenosis. Magli et al. published acceptable 1-year toxicity outcomes for a novel three-fraction regimen of SABR for low- and favorable intermediate-risk patients, to a dose of 40 Gy. MRI-based planning was utilized, gold fiducials and a hydrogel spacer were placed, and a catheter was inserted in the bladder for each fraction [15]. They observed rates of 11.9% and 1.7% acute grade 2 and 3 urinary toxicity, respectively, and 8.5% acute grade 2 rectal toxicity, all of which resolved by 12 months. Alayed et al. published safety and efficacy results of their prospective single-cohort study of low- and intermediate-risk patients undergoing a two-fraction SABR regimen to a dose of 26 Gy [16]. Radiation was delivered to the prostate alone using a 3 mm uniform expansion. The study was performed prior to development and standardization of peri-rectal hydrogel spacers. On their sample of 30 patients, they reported no acute grade 3 + GI or GU toxicity, and one instance each in later follow up, comparable or even slightly better than that from five-fraction protocols. An additional phase-II study has begun accruing of five vs. two fractions of SABR for low- and intermediate-risk prostate cancer (ClinicalTrials.gov identifier: NCT04984343), though requiring MRI-based adaptive therapy technology and utilizing lower radiation doses.

Multiple questions remain regarding the potential role for SABR in fewer than five fractions. These include optimal patient selection, ideal dose fractionation, proper techniques to assure necessary avoidance of surrounding normal tissue, and how to balance dose to organs at risk with the accepted requirement of a planning target volume (PTV) to account for inevitable setup uncertainty. Importantly, recent endoscopic reports reveal notably high rates of rectal ulceration after dose-escalated SABR [17], and placement of a hydrogel spacer has been demonstrated to significantly reduce this risk [18]. Placement of a resorbable hydrogel spacer to displace the rectum away from the high dose region, allowing for a posterior safety margin to account for potential intra-fractional motion, instead of a rectal balloon, is essential for accomplishing rectal sparing while facilitating additional assurance regarding treatment accuracy. A degree of posterior planning margin importantly helps assure adequate coverage of the posterior aspect of the prostate, coverage of which has been shows to correlate directly with tumor marker recurrence [19].

In the context of modern dose escalation, and in lieu of a strong body of HDR-brachytherapy data supporting a two-fraction approach to ultra-high dose treatment with initial experiences of two-fraction SABR, direct comparison of a non-invasive and broadly generalizable two-fraction SABR regimen to the standard approach of five-fractions is logical. Institutional studies have emerged in the recent years demonstrating evidence for safety of ultra-hypofractionated regimen in fewer than five fractions of SABR, and large-scale randomized data is much needed. Aided by the ability to achieve necessary rectal dose constraints through placement of a rectal hydrogel, this phase II/III prospective study will, with power to establish non-inferiority, compare standard 5-fraction SABR with a novel 2-fraction regimen.

## Methods/design

### Objective

The objective of this trial is to assess the oncologic and quality of life non-inferiority of 2-fraction SABR to 27 Gy compared to 5-fraction SABR to 40 Gy, in low- and favorable intermediate-risk prostate cancer.

### Study design

**Fig. 1:**
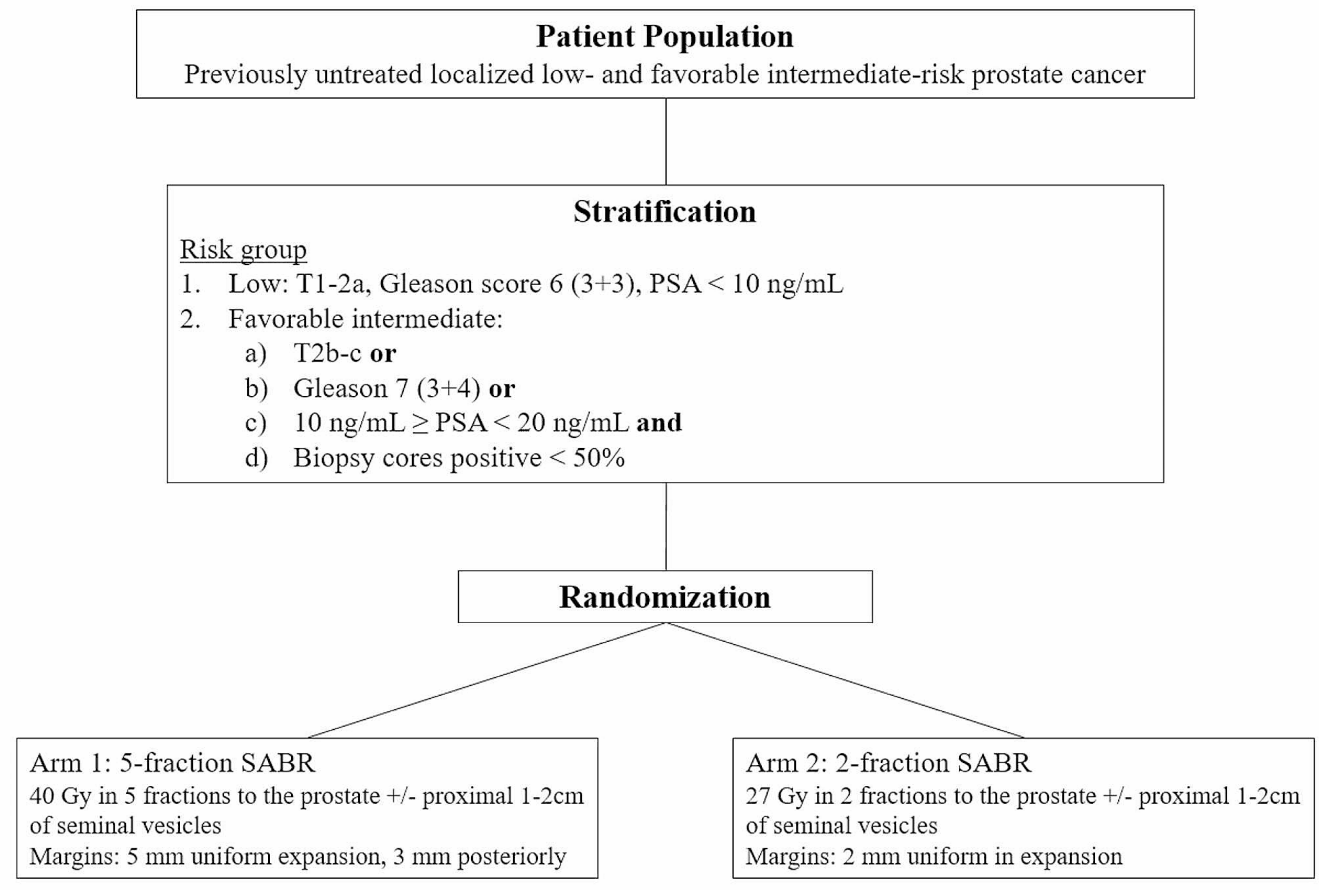
Schema of the SABR-Dual clinical trial

This is a prospective randomized phase II/III non-inferiority control trial with pre-planned interim analyses for safety signals in the 2-fraction arm. Participating centers will be tertiary, academic hospitals in Israel (updated list available at health.gov.il) with the potential of opening additional sites (limited to the United States and Canada) over the trial period. All aspects of the trial have been approved by the relevant institutional internal review boards. Subjects will be randomized between the standard arm of prostate SABR to 40 Gy in 5 fractions and the experimental regimen of 27 Gy in 2 fractions (Fig. 1). A focal simultaneous integrated boost (SIB) to the MRI-defined dominant intraprostatic lesion (DIL) will be permitted. All patients will undergo trans-rectal ultrasound-guided placement of a radiopaque hydrogel spacer and three gold fiducial intraprostatic markers prior to simulation. Both simulation CT and a planning MRI will be used for target and normal structure delineation, though SABR delivery will not require an MRI-guided linear accelerator (LINAC).

#### Endpoints

**Primary endpoint**.

1. Freedom from disease progression (FFDP), defined as clinical failure or biochemical failure per the Phoenix definition [20].

**Secondary endpoints**.

1. Progression free survival (PFS), defined as biochemical failure, clinical failure, or death from any cause.
2. Distant metastasis free survival [21].
3. Prostate cancer specific survival (PCSS).
4. Longitudinal PSA response.
5. Time to salvage treatment.
6. Physician-reported GU/GI toxicity at 1, 3, 6, 9, 12, 18, 24-months (CTCAE).
7. Patient-reported GU/GI toxicity at 1, 3, 6, 9, 12, 18, 24-months (IPSS, EPIC, SHIM).

#### Patient selection

**Inclusion criteria**.

- Male patients ≥ 18 years.
- Diagnosis of low- or favorable intermediate-risk prostate adenocarcinoma.
  - T1-T2c
  - PSA < 20
  - Gleason 6 or 7 (3 + 4)
  - Cannot had multiple intermediate-risk factors consistent with unfavorable-intermediate risk disease

- Prostate gland < 80 cc.
- IPSS < 15 (unaided by a-adrenergic inhibitor or anticholinergic drugs).

**Exclusion criteria**.

- Unfavorable intermediate-risk disease and above.
- Chronic inflammatory bowel condition (IBD, Crohn’s disease, Sarcoidosis, Rheumatic disease).
- Chronic immunosuppression.
- Contraindications to hydrogel spacer placement.
- Contraindications to a prostate MRI.
- Any prior prostate cancer treatment.
- Prior pelvic radiotherapy.
- Previous transurethral resection of the prostate (TURP) within 12 months.
- Hip prosthesis.
- Prior use of androgen deprivation therapy (ADT).

#### Pretreatment evaluation

Patients will undergo prostate cancer diagnostic evaluation with at least a standard whole-gland sextant biopsy to establish Gleason score. MRI-guided targeted biopsy is allowed as well but comprehensive whole-gland biopsy is required. An updated staging PSA will be obtained within 1 month prior to enrollment.

All patients are required to undergo a diagnostic multiparametric MRI (mpMRI) with 3T coil to identify regions suggestive of high-grade disease and to radiographically rule out the likelihood of extra-prostatic extension and/or seminal vesicle (SV) involvement. As eligible patients will have a categorization of low-risk or favorable intermediate-risk disease, systemic staging with a CT, bone scan, and/or PSMA-PET is allowed but will not be mandated.

Prior to enrollment patients will undergo a general physical exam. Baseline urinary, sexual, and bowel symptoms will be quantified using the International Prostate Symptom Score (IPSS), Sexual Health in Men (SHIM) score, and the global quality of life measure EPIC-26.

#### Data collection

All data collected on study will be entered electronically into a customized REDCap^™^ database and monitored by representative members of the Department of Clinical Research of Davidoff Cancer Center, Rabin Medical Center. All data will be retained through final censoring and follow up period, and for an additional five years thereafter. Following initial publication of clinical outcomes, deidentified data will be made available for review upon request.

### Treatment plan

#### Consent process

Written informed consent will be obtained from all subjects prior to enrollment by the treating physician.

#### Pre-simulation

Prior to simulation, all participants will undergo peri-rectal radiopaque hydrogel spacer insertion, as well as placement of 3–4 gold fiducial markers, to displace the rectum away from the high-dose radiation field and aid in target localization, respectively. If a patient is being treated using an MR-LINAC, fiducial markers will not be required.

#### Simulation imaging and immobilization

A treatment planning CT in the supine position will be acquired no sooner than in the week following hydrogel spacer placement, with the patient set up in the same position as for daily treatments, using appropriate hip, knee, and ankle support, with slice thickness of 1–2 mm. A treatment planning MRI (3-Tesla, minimum T2w sequence) will be acquired in a best approximation of the treatment position. Patients will be instructed to begin a daily stool softer beginning 1 week prior to hydrogel placement throughout the course of SABR, and will utilize a rectal enema before CT simulation and each fraction of radiation [22].

A full bladder is required for treatment with a consistent degree of bladder fullness determined by cone-beam CT (CBCT) or pre-treatment bladder scan. An empty rectum is required, and patients will be instructed to continue use of a prescribed stool softener throughout the course of SABR and place a rectal enema prior to each fraction.

#### MRI-CT registration

MRI-CT registration with a T2w MRI sequence serving as the primary fusion sequence will consist of a prostate-to-prostate registration. Precise fusion will be aided by prostate anatomy, fiducial markers, and hydrogel spacer. Internal peer review and approval of registration quality is strongly recommended. Where MRI-based treatment delivery is available, planning may be performed based on MRI alone.

#### Treatment technologies

Patients will be treated with photon-based radiation, IMRT/VMAT techniques. Allowed photon energies are 6–10 MV. Flattening filter free delivery is allowed.

#### Target volumes and margins

Gross tumor volume (GTV) will be defined as an MRI-visible PIRADS 4–5 lesions that is concordant with biopsy results. If no such lesion is visible, a GTV will not be included.

The clinical target volume (CTV) will be defined as the entire prostate gland, including GTV, as well as 1–2 cm of the seminal vesicles (SV) in the setting of favorable intermediate-risk disease.

A planning target volume (PTV) expansion will be added to account for possible variability in daily treatment set up and internal organ motion. The PTV margin will be dictated as 5 mm circumferential except for 3 mm posterior margin in the 5-fraction arm, and a uniform 2 mm expansion in the 2-fraction arm.

#### Radiation dose

Standard arm: Radiation dose will be 40 Gy divided into five equal fractions of 8 Gy per fraction to the prostate volume. When the proximal SV is included, the PTV volume of the SV that is beyond the PTV prostate volume will be prescribed 35 Gy, 7 Gy per fraction. When present, the GTV will receive an SIB to 45 Gy.

Experimental arm: Radiation dose will be 27 Gy divided into two equal fractions of 13.5 Gy per fraction to the prostate volume. When the proximal SV is included, the PTV volume of the SV that is beyond the PTV prostate volume will be prescribed 23 Gy, 11.5 Gy per fraction. When present, the GTV will receive an SIB to 30 Gy.

#### Treatment planning

In addition to the GTV, CTV, and PTV targets described above, the following organs at risk (OARs) will be contoured: bladder and bladder wall, rectum and rectal wall, urethra (with an additional 2 mm PRV avoidance structure uniformly expanded), femoral heads, large bowel, small bowel (where relevant), penile bulb, and proximal crura [23]. It is recommended to contour the fiducial markers and radiopaque hydrogel spacer to aid in daily setup. Target coverage goals are listed in Table 1 and OAR constraint goals for the standard and experimental arms are listed in Tables 2 and 3, respectively. Dose constraints may not be exceeded under any circumstance.

**Table 1:** Target volume goals for standard and experimental arms

Structure	Parameter (Gy)	Per protocol	Variation acceptable	Notes
PTV_4000	V40	≥ 95%		Arm 1
	V39.6	≥ 97%	≥ 95%	
	D0.03 cc*	< 107%	< 108%	
CTV_4000	V39.6	≥ 100%	≥ 99%	
PTV_3500	V35	≥ 95%		
CTV_3500	V33.95	≥ 97%	≥ 95%	
PTV_2700	V27	≥ 95%		Arm 2
	V26.73	≥ 97%	≥ 95%	
	D0.03 cc*	< 107%	< 108%	
CTV_2700	V26.73	≥ 100%	≥ 99%	
PTV_2300	V23	≥ 95%		
CTV_2300	V22.31	≥ 97%	≥ 95%	

*When a simultaneous integrated boost is prescribed, maximum allowed dose to the primary PTV (either PTV_4000 or PTV_2700) will be limited to 102% of the boost dose– D0.03cc < 45.9 Gy or D0.03cc < 30.6 Gy. There is no specific minimum required SIB coverage for GTV, when present, but minimal coverage of 95% of volume covered by 95% of dose is recommended.

**Table 2:** Organ at risk constraints for the standard arm

Structure	Parameter (Gy)	Per protocol	Variation acceptable
Bladder	D0.03 cc	< 42 Gy	D0.5cc < 41.2 Gy
	V26	≤ 25%	
	V26	≤ 50 cc	
Rectum	D0.03 cc	< 40.8 Gy	
	D1cc	< 38.5 Gy	D2cc < 38 Gy
	V36	≤ 10%	
	V32	≤ 20%	
	V20	≤ 50%	
Rectal wall (any slice)	35% circumf.	< 39 Gy	Semi-quantitative visualization
	50% circumf.	< 24 Gy	
UrethraPRV	D0.03 cc	< 40.8 Gy	
Urethra	D0.03 cc	≤ 40 Gy	
Femoral head	V22	≤ 10 cc	
	V22	≤ 5%	
Penile bulb	D0.03 cc	< 38 Gy	
	V22	≤ 30%	
Crura	D_mean_	< 4.7 Gy	
	D_2%_	< 12 Gy	
Skin	D0.03 cc	< 25 Gy	

**Table 3:** Organ at risk constraints for the experimental arm

Structure	Parameter (Gy)	Per protocol	Variation acceptable
Bladder	D0.03 cc	< 28.08 Gy	D0.5cc < 27.54 Gy
	V16.2	≤ 25%	
	V16.2	≤ 50 cc	
Rectum	D0.03 cc	< 27 Gy	
	D1cc	< 22.95 Gy	D2cc < 21.6 Gy
	V23	≤ 10%	
	V19	≤ 20%	
	V10	≤ 50%	
Rectal wall (any slice)	35% circumf.	< 20 Gy	Semi-quantitative visualization
	50% circumf.	< 13.5 Gy	
UrethraPRV	D0.03 cc	< 27.54 Gy	
Urethra	D0.03 cc	≤ 27 Gy	
Femoral head	V13.5	≤ 10 cc	
	V13.5	≤ 5%	
Penile bulb	D0.03 cc	< 24.3 Gy	
	V13.5	≤ 30%	
Crura	D_mean_	< 3.78 Gy	
	D_2%_	< 8.8 Gy	
Skin	D0.03 cc	< 13.5 Gy	

#### Quality assurance

Each treatment plan must be peer-reviewed by another attending radiation oncologist or at chart-review rounds, with confirmation of appropriate target coverage and OAR avoidance. Plans will also undergo a second quantitative review by a radiation physicist.

#### Treatment delivery

Radiation treatment must begin within 3 weeks after simulation. A single dose of 2 mg Dexamethasone will be given prior to each radiation treatment. A CBCT will be acquired prior to the start of each treatment and alignment to fiducial markers, hydrogel spacer, and overall PTV will be confirmed. Up to 2 mm and/or 2-degree corrections will be allowed without necessitating repeat imaging. Larger corrections will require repeat CBCT confirmation. The presence of a consistently full bladder and empty rectum will be checked. In both arms, a second CBCT will be acquired to re-confirm precise alignment after approximately half of the fractional dose is delivered (if the fraction is split into two arcs, the CBCT will be repeated in between the arcs). Real-time tracking of gold fiducials markers (where present) will also be utilized where available, in which case an additional CBCT is not necessary.

For patients not yet taking Tamsulosin, a dose of 0.4 mg will be prescribed prophylactically starting day 1 of radiation through 30 days following completion of radiation. For patients already taking 0.4 mg, the dose will be increased to 0.8 mg for the same period.

In the standard arm, fractions will be delivered every-other-day with a total treatment time of maximum two weeks. In the experimental arm, an inter-fraction interval of at least three days is required, and treatment will be completed within a maximum of seven days.

#### Patient monitoring

Baseline assessment will include PSA, quantitative urinary function (IPSS), sexual health (SHIM) score, and a global quality of life measure (EPIC-26). Follow up will occur at 1 month, 3 months, continuing at 3-month intervals through 1 year, then every 6 months from years 2–5, followed by annually thereafter. PSA will be recorded in follow up at 3, 6, 9, and 12 months in the first year, then every 6 months thereafter through five years, then annually. IPSS, SHIM and EPIC scores will be recorded during each follow up.

Adverse events (AE) will be described using the grading scales found in the revised NCI Common Terminology Criteria for Adverse Events (CTCAE), version 5.0. An AE is defined as any untoward medical occurrence associated with the use of a medical intervention, which in the context of this trial include radiopaque hydrogel spacer and fiducial marker placement, and radiation therapy. The following AEs should be assessed and recorded at each protocol mandated data collection interval: dysuria, hematuria, incontinence, urinary retention, diarrhea, rectal hemorrhage, fatigue. Full schedule of patient monitoring is summarized in Table 4.

#### Imaging and pathology surveillance

At two years following treatment, when feasible subjects will undergo a surveillance 3T MRI using multiparametric sequencing, followed by a combination MRI-guided biopsy and systematic whole gland biopsy (where feasible) for identification of any residual viable tumor. MRI findings and pathology will be compared between groups. If a patient is found to have a biopsy positive for residual adenocarcinoma, additional follow up and possible treatment will be discussed per physician assessment and patient preference.

**Table 4:** Schedule of patient assessments through the study period

Schedule of Assessments							Notes
Visit Name	SCR	Simulation & treatment	2-week toxicity assessment^a^	1st year follow up^b^	Follow up years 2-5^c^	Follow up year 6 and thereafter^d^	
Medical history	X						
Concomitant medication review	X						
Adverse events assessment			X	X	X	X	
Physical examination	X			X	X	X	
PSA	X			X	X	X	Staging PSA will be obtained within 1 month prior to enrollment
IPSS	X			X	X	X	
EPIC	X			X	X	X	
SHIM	X			X	X	X	
Hydrogel spacer insertion	X						
Staging PET CT/CT/Bone scan	X						optional
Prostate MRI	X				X		2-year MRI per physician recommendation
Prostate biopsy					X		Following the 2-year MRI– biopsy should be both targeted and systematic

a. For the first three patients. b. During the 1st -year, follow up will be performed at 1, 3, 6, 9, and 12 months. c. Every 6 months. d. Annually

#### Discontinuation/withdrawal

Subjects may voluntarily discontinue participation at any time. If a subject discontinues participation from the study, any remaining clinical and laboratory evaluations that would have been performed at what would have been the next scheduled follow up, such as PSA and quality of life questionnaires, will be obtained at that time. Any patient who may suffer an adverse event will remain in close medical follow up as long as deemed appropriate.

### Statistics and sample size calculation

#### Randomization

The study will utilize a 1:1 randomization between Arm 1: Arm 2, based on the stratification factors described in the Methods section. Randomization will take place in permuted blocks, details of block size known exclusively by the study statistician. The randomization sequence will be uploaded into a restricted-access database (REDCap), details of which known only to the statistician. The database is housed on secure hospital servers at Davidoff Cancer Center. Once a patient is enrolled, the database will be accessed by the primary trial coordinator to obtain the appropriate intervention in sequence, which will then be assigned to the patient.

#### Sample size

Target accrual will be set at 456, 228 in each arm, derived based on an assumption of 5-year bPFS of 90–91% with 5-fraction SABR, and designed to establish with 80% power and an α of 0.05 that 2-fraction SABR results in a 5-year bPFS that is not lower than the standard 5-fraction regimen by more than 7%. The noninferiority margin was chosen to be approximately one half of the absolute difference in 5-year bPFS observed among contemporary superiority trials of dose escalation (15–16%) in similar risk patients. Under assumed failure rates and guarding against dropout, the final targeted accrual will be 502 patients.

#### Analysis plan

The primary endpoint of this study is failure from disease progression (FFDP), defined as clinical or biochemical disease recurrence (biochemical failure as per PSA nadir + 2 ng/mL), excluding death. Death with no evidence of progression will be treated as a competing risk in the survival models. This study will utilize both an intent-to-treat and per-protocol analysis of the primary and secondary endpoints, with time-to event duration originating at random assignment. Prognostic characteristics, treatment delivery, and toxicity will be reported using descriptive statistics. Time-to-event endpoints will be calculated using the Kaplan-Meier method [24] and compared using the log-rank test. HRs with 95% CI will be computed using the Cox proportional hazards regression model for the end point–specific hazard. Frequency distributions of grade (0 to 5) for selected adverse events will be compared using x^2^ tests. 2 × 2 sub-tables will be formed to evaluate the differences in risk of grade 2 or 3 events, and relative risk (RR) estimates with 95% CIs will be computed. The definitive analysis is planned to occur at 5 years, with two planned interim safety analysis at mean follow up of one and two years.

Secondary endpoints (unpowered) will include progression free survival (PFS), distant metastasis-free survival (DMFS), prostate cancer-specific survival (PCSS), longitudinal PSA response, and time to salvage treatment, also assessed at five years. Both patient-reported and physician-reported treatment related adverse effects at six months, one, and two years will be compared and reported. In the setting of multiple secondary endpoints, the Benjamini-Hochberg FDR correction procedure will be applied. Treatment related AE will be primarily objectively measured using the EPIC-26 evaluation. MIDs for each of the domains will be set as follows: 5 for bowel and hormonal, 6 for urinary obstructive, 11 for sexual, and 8 for urinary incontinence [25].

### Data and safety monitoring committee

A predefined international independent data and safety monitoring committee will evaluate the acute toxicity profiles of the first three patients treated with 2-fraction SABR for approval to proceed, and again after treatment of the first 20 patients. The committee will review the chart and treatment plant of any patient who experienced a grade 3, 4, or 5 toxicity, and can, at it’s discretion, recommend early closure of the trial, dose adjustments, or protocol amendment. The committee will meet annually after study initiation to review toxicity outcomes. If any grade 3–5 toxicity is reported, the committee will review the case to determine if such toxicity was related to treatment. The committee can, at its discretion, recommend cessation of the trial or exclusion of certain treatment sites and/or delivery techniques that are deemed as high-risk for complications.

### Confidentiality of subject records

All personal healthy information of study participants will be kept de-identified and confidential. Identification of subjects will be through a numbering system coded uniquely for this trial. The subject identifier list matching participants with their code will be kept confidential by the principal investigator and lead trial coordinator. No public reports will contain any identifying information of any individual patient.

### Protocol amendments

The trial protocol will be amended if deemed necessary only through direct approval of the principal investigator, who will then be responsible to assure that changes are known to all co-investigators. Any amendments must be approved by the IRB committee of the primary trial institution, Rabin Medical Center. Abstract and manuscript authorship will be approved by the principal investigator at the time of submission.

## Discussion

This trial will be the largest and optimally powered prospective study to date investigating the safety, efficacy, and non-inferiority of definitive prostate SABR in just two fractions for low- and favorable intermediate-risk prostate cancer, compared to the current ultrahypofractionated standard regimen of five fractions. The potential implications of demonstrating non-inferiority of this regimen can influence practice for patients around the world.

While SABR in fewer than five fractions to the entire prostate has not yet been prospectively studied on this scale, there have been multiple phase I and II experiences published to date, supporting it’s essential safety. Kawakami et al. published a phase II series of 55 patients who received SABR in four fractions (equivalent BED to 40 Gy in five fractions), demonstrating reasonable efficacy [26]. While they reported a slight increase in toxicity, they did not use peri-rectal spacers, and also included high risk patients, some of whom had T3 disease, and allowed for the potentially confounding factor of hormone therapy. A novel three fraction regimen in a similar population of 59 patients, wherein fiducial markers and a hydrogel spacer were used, showed acceptable acute and 1-year toxicity [15]. A 2-fraction regimen in a cohort of 30 patients was published in a similar population of low and intermediate risk patients, with a favorable toxicity profile at a mean follow up of 44 months [16]. This trial, however, required placement of an endorectal immobilization device for the simulation and each treatment. A recent multi-institutional phase I/II single arm study of single-fraction urethral sparing radiation in 45 patients also demonstrated promising short-term toxicity outcomes [27].

In contrast, in designing this trial, we intentionally selected an already established dose-fractionation from the 2-fraction radiation modality with the most substantial data, namely HDR-brachytherapy. 27 Gy given in two fractions is already known to be a highly safe and effective regimen in the setting of brachytherapy, with the downside of it comprising an invasive procedure under anesthesia performed twice. As such, of all the regimen thus far having begun to be studied, our dose in two fractions is rooted in many years of published data. A small phase II study of single-fraction SABR showed potentially similar toxicity outcomes in favorable intermediate-risk patients [14], but an instance of late high grade urethral stenosis, overall higher than expected toxicity, and a trend toward inferior PFS, supported not pursuing this method further.

A second important priority in the design of this trial is the goal of presenting a planning and treatment methodology that is reproducible and broadly generalizable. While MR-LINAC technology is emerging as an important tool with possible clinical advantages [28], it is not a widely available technology and in many instances cost-prohibitive. Adaptive radiotherapy may offer similar benefits but also shares similar limitations. For these reasons, as well as to minimize patient discomfort during and surrounding treatment, we did not include endorectal applicators or immobilizers. Given the known importance of including a PTV expansion to account for minor variations in setup to assure accuracy of treatment, we did mandate insertion of a radiopaque hydrogel spacer, which has also been demonstrated on prospective studies to decrease the risk of long-term rectal toxicity by displacing the rectum away from the high dose field [29, 30].

Our study population was carefully selected to minimize the possible confounding of external factors to the oncologic and toxicity endpoints. While one could argue that it is clinically more meaningful to study radiation developments in a higher risk population, variation in target volumes, such as whether or not to include pelvic nodes, and the impact of variable duration.

ADT, could cloud the ability to more directly conclude the relative differences strictly between different radiation therapy regimen.

Thoroughly studying the potential of 2-fraction SABR as a new standard of care in the treatment of localized prostate cancer is the natural next step in a process that has been ongoing since the understanding emerged of the need for dose-escalated radiotherapy. We have witnessed the standard treatment regimen decrease from more than 40 fractions to 28, 20, to the 5–7 most commonly used in prostate SABR [9]. SABR-Dual represents a further decrease of these regimen of an additional 60–70%. In an ever-expanding global society with patients often traveling long distances for oncologic care, high-level treatment centers carrying large volumes of patients, and the worldwide ubiquity of prostate cancer, the SABR-Dual regimen, firmly based in both mature and more recent data, has the potential to offer substantial impactful benefits.

## Conclusion

SABR-Dual is a prospective randomized phase II/III study comparing standard 5-fraction SABR to a novel 2-fraction SABR regimen, appropriately powered to demonstrate non-inferiority. The 2-fraction dose is firmly rooted in a mature experience using HDR-brachytherapy. Radiation delivery on this study is accessible and widely generalizable as expensive and limited technologies such as MR-LINAC, adaptive planning, and daily rectal probe insertion are not mandated. Such treatment, if shown to be non-inferiorly safe and effective as standard 5-fraction SABR, may become a practice altering new standard of care in the treatment of low- and favorable intermediate-risk prostate cancer.

## Data Availability

Not applicable.

## Declarations

### Clinical trial registration

ClinicalTrials.gov Identifier: NCT06027892. Date of registration: 9/7/2023.

Health.gov.il identifier: MOH_2022-08-30_012007. Date of registration: 8/30/2022.

### Data sharing statement

Deidentified participant data from this trial will not be shared publicly, however, the full protocol will be published along with the primary analysis of the outcomes.

## References

[1] Dearnaley DP, Jovic G, Syndikus I, Khoo V, Cowan RA, Graham JD (2014). Escalated-dose versus control-dose conformal radiotherapy for prostate cancer: long-term results from the MRC RT01 randomised controlled trial. Lancet Oncol.

[2] Kuban DA, Levy LB, Cheung MR, Lee AK, Choi S, Frank S (2011). Long-term failure patterns and survival in a randomized dose-escalation trial for prostate cancer. Who dies of disease?. Int J Radiat Oncol Biol Phys.

[3] Michalski JM, Moughan J, Purdy J, Bosch W, Bruner DW, Bahary JP et al. Effect of Standard vs Dose-Escalated Radiation Therapy for Patients With Intermediate-Risk Prostate Cancer: The NRG Oncology RTOG 0126 Randomized Clinical Trial. JAMA Oncol. 2018; 14;4(6):e180039. 10.1001/jamaoncol.2018.0039. 10.1001/jamaoncol.2018.0039 PMC5885160 29543933

[4] Zietman AL, DeSilvio ML, Slater JD, Rossi CJ, Miller DW, Adams JA (2005). Comparison of conventional-dose vs high-dose conformal radiation therapy in clinically localized adenocarcinoma of the prostate: a randomized controlled trial. JAMA.

[5] Michalski JM, Moughan J, Purdy J, Bruner DW, Amin M, Bahary JP (2023). Long-term outcomes of NRG/RTOG 0126, a Randomized Trial of High Dose (79.2 gy) vs. standard dose (70.2 gy) Radiation Therapy (RT) for men with localized prostate Cancer. Int J Radiat Oncol Biol Phys.

[6] Dearnaley D, Syndikus I, Mossop H, Khoo V, Birtle A, Bloomfield D (2016). Conventional versus hypofractionated high-dose intensity-modulated radiotherapy for prostate cancer: 5-year outcomes of the randomised, non-inferiority, phase 3 CHHiP trial. Lancet Oncol.

[7] Catton CN, Lukka H, Gu CS, Martin JM, Supiot S, Chung PWM (2017). Randomized Trial of a Hypofractionated Radiation Regimen for the treatment of localized prostate Cancer. J Clin Oncol.

[8] Lee WR, Dignam JJ, Amin MB, Bruner DW, Low DL, Swanson GP (2016). Randomized Phase III Noninferiority Study Comparing Two Radiotherapy Fractionation Schedules in patients with low-risk prostate Cancer. J Clin Oncol.

[9] Jackson WC, Silva J, Hartman HE, Dess RT, Kishan AU, Beeler WH (2019). Stereotactic body Radiation Therapy for localized prostate Cancer: a systematic review and Meta-analysis of over 6,000 patients treated on prospective studies. Int J Radiat Oncol Biol Phys.

[10] Hoffman KE, Penson DF, Zhao Z, Huang L, Conwill R, Laviana AA (2020). Patient-reported outcomes through 5 years for active surveillance, surgery, Brachytherapy, or External Beam Radiation with or without androgen deprivation therapy for localized prostate Cancer. JAMA.

[11] Pickles T, Morris WJ, Kattan MW, Yu C, Keyes M (2011). Comparative 5-year outcomes of brachytherapy and surgery for prostate cancer. Brachytherapy.

[12] Viani GA, Arruda CV, Pellizzon ACA, De Fendi LI (2021). HDR brachytherapy as monotherapy for prostate cancer: a systematic review with meta-analysis. Brachytherapy.

[13] Morton G, McGuffin M, Chung HT, Tseng CL, Helou J, Ravi A (2020). Prostate high dose-rate brachytherapy as monotherapy for low and intermediate risk prostate cancer: efficacy results from a randomized phase II clinical trial of one fraction of 19 gy or two fractions of 13.5 gy. Radiother Oncol.

[14] Greco C, Pares O, Pimentel N, Louro V, Santiago I, Vieira S (2021). Safety and Efficacy of virtual prostatectomy with single-dose radiotherapy in patients with intermediate-risk prostate Cancer: results from the PROSINT phase 2 Randomized Clinical Trial. JAMA Oncol.

[15] Magli A, Farneti A, Faiella A, Ferriero M, Landoni V, Giannarelli D (2021). Toxicity at 1 year after stereotactic body Radiation Therapy in 3 fractions for localized prostate Cancer. Int J Radiat Oncol Biol Phys.

[16] Alayed Y, Cheung P, Chu W, Chung H, Davidson M, Ravi A (2019). Two StereoTactic ablative radiotherapy treatments for localized prostate cancer (2STAR): results from a prospective clinical trial. Radiother Oncol.

[17] Kim DWN, Cho LC, Straka C, Christie A, Lotan Y, Pistenmaa D (2014). Predictors of rectal tolerance observed in a dose-escalated phase 1–2 trial of stereotactic body radiation therapy for prostate cancer. Int J Radiat Oncol Biol Phys.

[18] Folkert MR, Zelefsky MJ, Hannan R, Desai NB, Lotan Y, Laine AMA, Multi-Institutional (2021). Phase 2 trial of high-dose SAbR for prostate Cancer using rectal spacer. Int J Radiat Oncol Biol Phys.

[19] Marcello M, Denham JW, Kennedy A, Haworth A, Steigler A, Greer PB (2020). Reduced dose posterior to prostate correlates with increased PSA Progression in Voxel-Based analysis of 3 Randomized phase 3 trials. Int J Radiat Oncol Biol Phys.

[20] Abramowitz MC, Li T, Buyyounouski MK (2008). The Phoenix definition of biochemical failure predicts for overall survival in patients with prostate cancer. Cancer.

[21] Gharzai LA, Jiang R, Wallington D, Jones G, Birer S, Jairath N (2021). Intermediate clinical endpoints for surrogacy in localised prostate cancer: an aggregate meta-analysis. Lancet Oncol.

[22] Yahya S, Zarkar A, Southgate E, Nightingale P, Webster G (2013). Which bowel preparation is best? Comparison of a high-fibre diet leaflet, daily microenema and no preparation in prostate cancer patients treated with radical radiotherapy to assess the effect on planned target volume shifts due to rectal distension. Br J Radiol.

[23] Achard V, Zilli T, Lamanna G, Jorcano S, Bral S, Rubio C et al. Urethra-Sparing Prostate Cancer Stereotactic Body Radiation Therapy: Sexual Function and Radiation Dose to the Penile Bulb, the Crura, and the Internal Pudendal Arteries From a Randomized Phase 2 Trial. Int J Radiat Oncol Biol Phys. 2023:S0360-3016(23)08307-4. 10.1016/j.ijrobp.2023.12.037. 10.1016/j.ijrobp.2023.12.037 38160915

[24] Kaplan EL, Meier P, Kotz S, Johnson NL (1992). Nonparametric estimation from incomplete observations. Breakthroughs in statistics. Springer Series in statistics (perspectives in statistics).

[25] Skolarus TA, Dunn RL, Sanda MG, Chang P, Greenfield TK, Litwin MS (2015). Minimally important difference for the expanded prostate Cancer Index Composite Short Form. Urology.

[26] Kawakami S, Tsumura H, Satoh T, Tabata K, Sekiguchi A, Kainuma T (2022). A phase II trial of stereotactic body radiotherapy in 4 fractions for patients with localized prostate cancer. Radiat Oncol.

[27] Zilli T, Franzese C, Guckenberger M, Giaj-Levra N, Mach N, Koutsouvelis N (2024). ONE SHOT - single shot radiotherapy for localized prostate cancer: 18-month results of a single arm, multicenter phase I/II trial. Radiother Oncol.

[28] Kishan AU, Ma TM, Lamb JM, Casado M, Wilhalme H, Low DA (2023). Magnetic resonance imaging-guided vs computed tomography-guided stereotactic body radiotherapy for prostate Cancer: the MIRAGE Randomized Clinical Trial. JAMA Oncol.

[29] Folkert MR, Zelefsky MJ, Hannan R, Desai NB, Lotan Y, Laine AM (2021). A multi-institutional phase 2 trial of high-dose SAbR for prostate Cancer using rectal spacer. Int J Radiat Oncol Biol Phys.

[30] Armstrong N, Bahl A, Pinkawa M, Ryder S, Ahmadu C, Ross J (2021). SpaceOAR Hydrogel Spacer for reducing Radiation Toxicity during Radiotherapy for prostate Cancer. Syst Rev Urol.

